# Breaking the glass ceiling: An interview with Yuhao (Jet) Zhou, María Óskarsdóttir, and Cristián Bravo

**DOI:** 10.1016/j.patter.2026.101541

**Published:** 2026-04-10

**Authors:** Yuhao Zhou, María Óskarsdóttir, Cristián Bravo

## Abstract

Despite years of attention to gender equality, women remain underrepresented on corporate boards. Zhou et al. explore how professional connections influence access to these positions and the reasons behind the underrepresentation.^1^ In this interview, three authors reflect on the inspiration behind their research, the teamwork that shaped the project, and the key ideas underlying their analysis of gender disparities on corporate boards.

## Main text

### What would you like to share about your background?

Yuhao (Jet) Zhou (JZ): I was trained in quantitative and corporate finance with a strong foundation in statistical modeling and risk analytics. From early on, I was interested in both the theoretical and practical sides of finance. Over time, my interests expanded toward network science and machine learning, particularly in understanding how network data can enhance analysis in corporate governance and financial decision-making. Professionally, I currently work in enterprise-level model risk management in the Canadian banking sector.

María Óskarsdóttir (MO): I am from Iceland, where gender equality is part of the national identity, so to me, it really is a fundamental right. I started my academic journey in mathematics, which then led me to do a PhD in applied economics. I held an academic position in the Department of Computer Science at Reykjavik University in Iceland and am now a Lecturer of Mathematical Modelling at the University of Southampton. I am not too fond of labels and find it a bit difficult to answer when I am asked what discipline I belong to. My research has a common factor, though: I use and develop AI methods, including deep learning, to address societal challenges and model complex economic and social systems, such as the corporate social networks in this paper.

Cristián Bravo (CB): I have the privilege to run a research center full of some of the brightest persons I have ever had the pleasure of working with. If I can say something about my lab’s ethos it is that we cover the full spectrum of AI in banking, from theory to deployment. This ethos has allowed us to work in challenging theoretical problems, applied research with some of the top companies and government organizations worldwide, and as is the case in this paper, using AI to tackle some of the core societal issues that exist within financial institutions. I am truly fortunate to lead such an excellent team and to be able to work in such stimulating and challenging problems.

### What is the definition of data science in your opinion? What is a data scientist? Do you self-identify as one?

JZ: To me, data science begins with letting the data speak first. It is a data-oriented process of distinguishing signal from noise through statistical reasoning, computational tools, and domain knowledge. Credible insight requires clarity about the model’s purpose, careful attention to data quality, methodological soundness, and rigorous testing before interpretation.

A data scientist is not simply someone who builds predictive models. It is someone who understands how data are generated, evaluates underlying assumptions, and ensures that conclusions are supported by evidence rather than narrative. I do identify as a data scientist, particularly in contexts where analytical rigor and responsible interpretation are as important as predictive performance.

### What drew you to this area of research? How has the research focus of your team evolved over the years?

CB: This idea was born 11 years ago! While the core research of my lab is AI in banking, I had seen this topic come repeatedly in specialized media, and other research had shown the board composition had an impact in financial performance. The first studies that showed that companies with a more diverse board of directors performed better were being published, and a seminal paper that showed that the size of the Rolodex of a director had a meaningful impact on a company’s performance had appeared.^2^ At this time, we had published our first paper on the use of social networks for measuring individual fraud risk.^3^ What made our techniques different from standard econometric models was that we could track individual performance, at the cost of strict causality. I knew the results would be useful, but the science wasn’t there yet to allow us to model career trajectories using detailed, individual-level information across multiple networks without having to construct features ourselves, thus losing many of the nuances that AI can bring. We (and many other researchers worldwide) kept working at developing new methodologies, such as our work on the propagation of risk in complex, dynamic networks.^4^ At this stage, we had everything we needed, so the idea could finally come to fruition. One thing I love of this specific paper is that it followed closely how AI, and AI in the financial sector, has evolved and what questions it can help answer.

### JZ, what drew you to your current team and topic? Was there a particular element that motivated you to participate in this project?

JZ: The project was shaped both by prior research and by collaboration. Influential work such as Fracassi and Tate^5^ highlighted that directors’ external networking can shape governance dynamics within firms with downstream implications for firm performance and value. This insight suggested that relational structure is not merely a sociological concept but an economically meaningful and measurable feature of governance systems. That raised a natural question: if networks matter, can we measure their structure more formally and integrate them systematically into empirical analysis?

At the same time, collaborating with Prof. María Óskarsdóttir, whose expertise lies in network science, played a crucial role. Her structural approach encouraged a more formal treatment of relational data and helped frame governance questions in terms of measurable network drivers. She also brought a clear perspective on gender and leadership disparities, which deepened and enriched the paper by connecting structural analysis to substantive questions about access and representation. Together, this perspective suggested that governance research could move beyond static measures of board composition toward formally modeling how relational structure shapes opportunities and disparities within corporate governance.

The combination of theoretical motivation and interdisciplinary collaboration ultimately shaped the direction and the scope of the project.

### Aside from supervising their research, CB, how do you help to develop and mentor your students and postdocs as scientists?

CB: As an applied researcher in a topic with strong demand, the greatest challenge to me when supporting the young researchers who choose my lab is to provide the complete set of skills that they will need to be successful both in academia and in industry. Industry is absorbing most of my graduates, which I see as a great thing, given the higher salaries normally offered and the opportunity to create impact at different levels (academia, industry, and government). I have worked to devise a training plan that includes the ability to develop independent research, and an independent research line, which is the basic skill we need to provide. I have also strived to provide the skills to be independent in planning and executing research plans with minimum oversight, how to participate and work in industry and government projects with deliverables (which I am fortunate to be able to do given the strong collaborations we have), how to network and create solid collaboration strategies that accompany you in your future career (this paper is a great example of that), and finally, how to work within the grant environment and secure the funds necessary to conduct research. Last but not least is the training required to conduct safe AI. Everyone in my lab receives training on data security, ethics, and understanding the impact of our work in society. I strongly believe it is what is needed to work with the powerful models we can deploy, which affect in a very real way the life of citizens.

### How did you come to collaborate?

CB: I am very fortunate to have written this paper with two great young researchers, and two of my closest collaborators, who also became, in time, some of my closest friends. Prof. Davison met me when I was on my PhD and was invited to give a talk in my home country (Chile), and who later invited me to apply to the Canada Research Chair position I now hold. When Jet applied to our PhD, we chose to co-supervise him to continue a successful collaboration. With Prof. María Óskarsdóttir, I had the fortune to be a part of her own supervisory committee, after her main supervisor, the late Prof. Bart Baesens, invited me to participate after we had published a very successful paper in network science.^3^ María specialized in network science applied to finance, so continuing the collaboration has been natural. We have published together many papers now, plus a book that came out recently,^6^ supervised several PhD students together, and participated in research projects with private and public funds. I can say it has been the most successful collaboration of my career.

### How does your collaboration work logistically? What are the challenges of collaboration between teams in different countries?

MO: With modern technology, distance in both time and space has decreased, making collaboration across countries and continents highly efficient and effective. However, it can be challenging to work around the time difference, especially when we all have busy schedules, as I am based in Europe and the rest of the team in Canada, but I think that because we all saw the value and importance of the research, we were willing to make it work. Although the research can all be done remotely, meeting in person is extremely valuable as there is an incentive to make the most of the time and work more intensively. In this collaboration, it actually proved very effective. I was in Canada when the data collection was taking off and also in the early stages of the modeling part. Jet also came to Iceland for a research stay with me, and then we all met twice at conferences. Each time we met, the project took a big leap forward as we could brainstorm, discuss ideas, and try them out with more ease. That can be more difficult when working at a distance.

### How important was the collaboration to the success of the paper? How important do you think collaboration is in general to research?

MO: The collaboration was crucial to the paper’s success as we brought complementary skills and expertise to the project. We come from very different backgrounds and therefore have different points of view. This was extremely valuable for the project as we were studying people from different backgrounds and therefore our own experiences were important when making sense of the results.

In general, I think that collaboration in research is extremely important. Collaboration means greater access to resources, skills, and expertise but also different points of view, which makes the work less biased and more mindful and inclusive. When doing research, we stand on the shoulders of giants. We learn by collaborating with those who came before us. Through collaboration, we pass that knowledge on. In particular, in interdisciplinary projects, it is vital to involve people from other disciplines as each one will provide a depth of understanding of the problems, the methods, the results, and how to interpret them. As a result, we can understand the world a little bit better, which is what research is about.

### Talking about your *Patterns* paper, what originally motivated you to study the link between professional networks and gender disparities in corporate board appointments?

JZ: I was interested in whether professional networks could be formally measured and analyzed alongside traditional qualifications such as experience and performance. While board appointments are shaped by many factors, prior research suggests that social capital also plays a role. I wanted to understand how much network position matters once observable characteristics are accounted for. Viewing this question through the lens of gender disparities made it even more compelling. If access to leadership depends partly on social capital, then differences in network opportunities may help explain gaps that are not visible in experience and performance alone.

### In simple terms, what is the most important message you want business leaders and policymakers to take away from this study?

CB: To allow our top female talent to get to the positions they can excel at, we need to support them throughout their careers. Our paper^1^ shows that more is demanded of women directors than their male counterparts, and all the networks they build across their careers are relevant to achieve elite positions. While hopefully this gender inequity will in time equalize as more women access these elite positions, the reality today is that their school, professional, and even charitable or social networks can have an outsized impact in their ability to reach the upper echelons of management. This means that universities need to foment clubs where these networks are created and talent management programs within companies can be crafted, understanding that the connections they create will have impact long after the employees have departed. If these programs are designed with the vision that they are supporting their members for decades to come, the momentum necessary to improve equality of opportunity in the field will grow even more than today.

### Did you encounter any particular difficulties, or were there any specific challenges about data, data management, or FAIR data sharing that you dealt with? How did you overcome them? Can others use the solutions you used to overcome these challenges?

JZ: Yes, there were several practical challenges. The data span multiple years and involve constructing longitudinal professional networks, which required extensive cleaning, standardization, and validation to ensure consistency over time. Career histories are highly heterogeneous with non-standardized job titles that had to be systematically categorized before analysis.

Building the final database from raw records took nearly a year of dedicated data construction and validation. The project ultimately captured decades of director and executive histories across thousands of publicly traded firms, resulting in tens of millions of professional connections. Transforming these raw records into a coherent network dataset required substantial data engineering before any analytical modeling could ever begin.

Because the project relies on proprietary datasets, the raw data cannot be publicly shared. To address this limitation, we focused on methodological transparency—carefully documenting preprocessing steps, clearly describing the network construction process, and structuring the analysis pipeline so that readers can understand how the results were obtained.

### Looking ahead, how could data science and AI help reduce gender inequality in leadership roles?

MO: Data science and AI are great at detecting patterns in vast amounts of data and as such they can be used to study gender inequality from a historic point of view, but also in a forward-looking manner, to identify what has changed and also what has not changed. This will allow for the design of interventions informed by real data, which can then be better targeted to the actual problem and, hopefully, be part of the solution in reducing inequality. That does not have to be limited to gender as inequality appears in other forms too.

**Figure:**
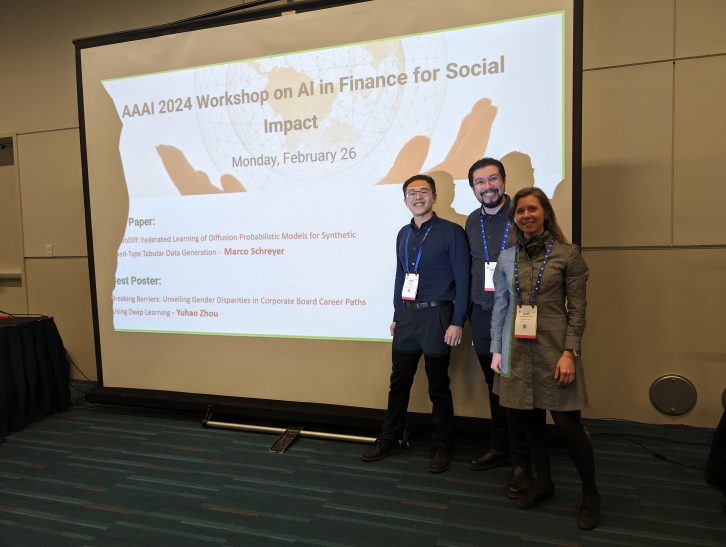
Presentation at AAAI conference workshop (Jet Zhou, Cristián Bravo, and María Óskarsdóttir)

## Acknowledgments:

J.Z. acknowledges the Ontario Graduate Scholarship program. J.Z. and C.B. acknowledge the support of the NSERC Discovery Grant program grants (RGPIN-2020-07114) and (RGPIN-2020-06667). C.B. also acknowledges the support of the SSHRC Insight program (435-2025-1038) and the Canada Research Chair program (CRC-2024-00192). M.O. acknowledges the support of the Icelandic Research Fund (IRF) (grant number 228511-051).
